# Dietary folate intake and serum klotho levels in adults aged 40–79 years: a cross-sectional study from the national health and nutrition examination survey 2007–2016

**DOI:** 10.3389/fnut.2024.1420087

**Published:** 2024-07-08

**Authors:** Yang Liu, Chunhuan Zhou, Rongjun Shen, Anxian Wang, Tingting Zhang, Zhengyuan Cao

**Affiliations:** ^1^Department of Medical Laboratory, Guihang 300 Hospital Affiliated to Zunyi Medical University, Guiyang, China; ^2^Hospital Infection Control Department, Guihang 300 Hospital Affiliated to Zunyi Medical University, Guiyang, China; ^3^Department of Endocrinology, Guihang 300 Hospital Affiliated to Zunyi Medical University, Guiyang, China

**Keywords:** klotho, folate, dietary, intake, aging, NHANES

## Abstract

**Objective:**

This study aims to explore the relationship between dietary folate intake and serum Klotho levels in adults from aged 40 to 79 years in the United States, seeking to elucidate the intricacies of their interaction.

**Methods:**

Analyzing data from the National Health and Nutrition Examination Survey (NHANES) spanning 2007 to 2016. The survey research determined folate intake through a 24-h dietary recall and nutrient density modeling, and assessed Klotho levels using enzyme-linked immunosorbent assay (ELISA). The relationship between folate intake and Klotho levels was evaluated using weighted linear regression, and complemented by analysis via smoothed curve models for nuanced understanding.

**Results:**

The study encompassed 10,278 participants, with an average age of 57.64 years, revealing a noteworthy positive correlation between dietary folate and serum Klotho levels. The regression coefficient stood at 0.11 (95% confidence interval, 0.05, 0.18) post-adjustment for various covariates. When dietary folate intake was categorized into quartiles, the second, third, and fourth quartiles exhibited statistically significant differences compared to the lowest quartile. This indicates that higher folate intake correlates with increased serum Klotho levels. These findings underscore the potential benefits of elevating folate intake to enhance serum Klotho levels. Stratified analysis indicated that this association was more pronounced among males aged 60 years or older and individuals with hypertension.

**Conclusion:**

The findings suggest a significant correlation between increased dietary folate intake and elevated serum Klotho levels in adults aged 40–79 years. Hinting at the potential nutritional influences on the aging process and associated health conditions. This calls for further exploration into the mechanisms and broader implications of this association.

## 1 Introduction

Aging is universally recognized as a multifaceted process influenced by an intricate interplay of genetic, environmental, and lifestyle determinants (1–3). This phenomenon spans a comprehensive spectrum of biological transformations, including diminished cellular functionality (4), the accumulation of DNA damage (5), and anomalies in amino acid metabolism (6). These transformations transcend singular biological levels, engaging in complex interactions that cumulatively precipitate the deterioration of tissue and organ function, thereby impacting the organism’s health and longevity. Consequently, it is crucial to thoroughly understand these processes. Effective interventions can slow down the aging process and enhance quality of life.

Since 1997, the Klotho protein has emerged as a focal point in anti-aging research (7, 8). It is predominantly expressed in the kidneys (9) and found in other tissues including the brain (10). Klotho plays a pivotal role in fostering longevity and health. It does so by modulating calcium and phosphorus metabolism, contributing to the antioxidant defense, and impacting critical cellular signaling pathways, notably Wnt/β-catenin and insulin-like growth factor-1 (IGF-1) (11, 12). A decline in Klotho gene expression is intricately linked to various aging-associated diseases, such as cardiovascular disease (13), osteoporosis (14), and chronic kidney disease (15). Research demonstrates that enhancing Klotho protein levels exogenously or activating its signaling pathways can mitigate these conditions and potentially slow the aging process (13, 16–19). Therefore, Klotho proteins not only serve as crucial markers for deciphering the mechanisms underlying aging, but also offer promising avenues for developing novel anti-aging therapies (8).

Folate, a critical component of the B-vitamin family, occupies a pivotal role in processes such as cell division and growth (20, 21), DNA synthesis and repair (22), and the metabolism of amino acids (23). Its significance is particularly pronounced during the initial stages of development, where it is indispensable in forestalling congenital disorders, including neural tube defects (24). Furthermore, a deficiency in folate intake can precipitate a range of health issues, encompassing anemia, cardiovascular diseases, and even cognitive impairments (25, 26). Ongoing scientific investigation continues to unveil the advantages of folate in supporting cardiovascular health, enhancing cognitive function, and contributing to anti-aging benefits (27). Therefore, it is essential to supplement folate for promoting health and preventing disease in pregnant women, those planning pregnancy, and individuals at risk of folate deficiency. However, high-dose supplementation is unnecessary for healthy individuals with normal folate levels (28).

Recent research has elucidated that particular dietary practices and the intake of specific types of food are closely linked to fluctuations in klotho protein levels (29, 30). Considering the pivotal roles that both klotho protein and folate occupy in fostering health and decelerating the aging process, understanding the dynamics of their interplay is of paramount importance.

## 2 Materials and methods

### 2.1 Study population

**Figure 1:**
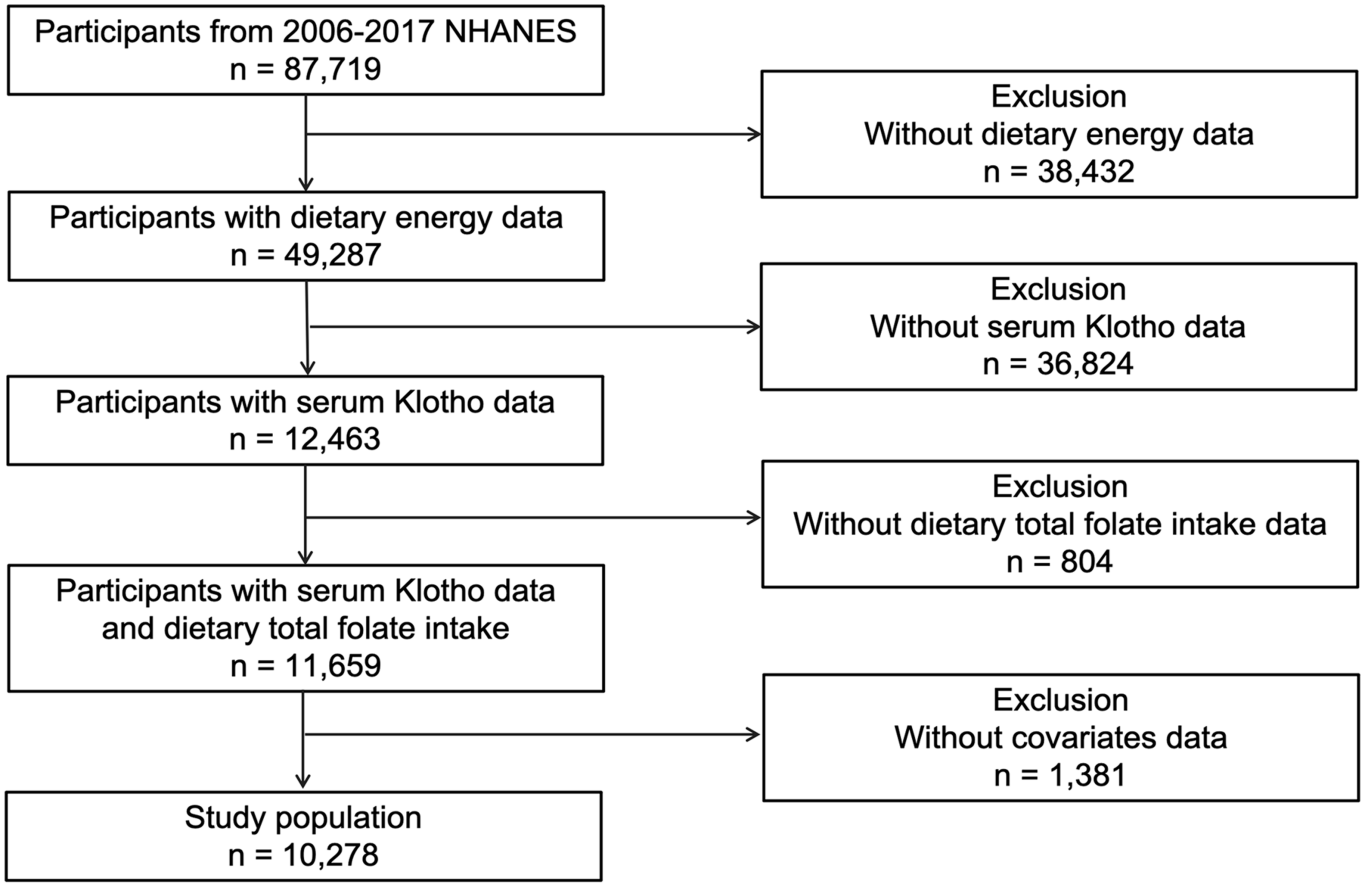
Flowchart of the study population.

This investigation drew upon data from five successive cycles of the National Health and Nutrition Examination Survey (NHANES) spanning from 2007 to 2016, encompassing 87,719 participants. Following the exclusion of subjects due to incomplete data on serum Klotho levels (*n* = 36,824), Dietary energy (*n* = 38,432), dietary folate intake (*n* = 804), and other covariates (*n* = 1,381). the study’s final cohort consisted of 10,278 individuals (Figure 1). The NHANES employed a sophisticated multistage probability sampling technique, orchestrated by the National Center for Health Statistics (NCHS). Informed Consent was signed by each participant, and the methodology of data collection received approval from an ethics committee, ensuring the protection of participants. Comprehensive details regarding the survey are accessible on the NHANES website.^1^

### 2.2 Measurement of dietary folate intake

In this study, the 24-h dietary recall was conducted using a combination of face-to-face and telephone interviews. The initial interview was carried out at the Mobile Examination Center (MEC), followed by subsequent interviews conducted via telephone 3 to 10 days later. This approach employed the USDA’s Automated Multiple-Pass Method (AMPM),^2^ which precisely recorded the types and quantities of foods and beverages consumed by participants within the 24 h prior to the interview. The nutrient content of these consumables was then assessed through a comprehensive dietary survey. The nutrient calculations used the USDA’s Food and Nutrient Database for Dietary Studies (FNDDS 4.1; see Footnote 2). Moreover, the average of the two dietary recalls was taken, reflecting only the actual intake levels, not the habitual intake of the population. To minimize the measurement errors associated with self-reported dietary assessment tools, we standardized nutrient intake relative to total energy intake and accounted for variations in individual energy needs. The adjusted intake formula is: dietary folate intake / energy intake * 1000 (31). This method, commonly referred to as nutrient density model, allows for a more precise evaluation of dietary folate intake across diverse energy requirements.

Additionally, in sensitivity analyses, we extended this method to assess folate from natural foods, folic acid from fortified foods and supplements, and dietary vitamin B12. Each nutrient’s intake was similarly adjusted for energy, ensuring robustness in our analyses across different dietary sources and intake patterns.

### 2.3 Serum klotho levels

Blood samples from participants were transported to the Northwest Laboratory for Lipid Metabolism and Diabetes Research, adhering to predefined protocols, and subsequently preserved at −80°C for analysis. The measurement of Klotho levels was conducted utilizing an ELISA kit produced (“IBL International,” Japan). In a bid to uphold laboratory standards and mitigate potential detection bias attributable to random variance, the samples underwent duplicate analyses. The mean of these analyses was accepted contingent upon compliance with the in-lot quality control criteria. Samples demonstrating a variance exceeding 10% between the two measurements were subjected to reevaluation and duly documented.

### 2.4 Assessment of covariates

The study incorporated a comprehensive set of covariates, totaling 11 which included both continuous and categorical variables. These covariates are as follows: sex (categorized as male or female), age, racial/ethnic background (categorized as non-Hispanic white, non-Hispanic black, Mexican American, or other, which includes multiracial and other Latino identities), marital status (categorized as married, not currently partnered, or cohabiting with a partner), and household income relative to the poverty threshold (categorized into three groups: ≤1.30, 1.31–3.50, and > 3.50 based on the poverty income ratio [PIR]). The poor income ratio was determined based on Federal Poverty Level (FPL) information, which considers factors such as inflation and family size.^3^ The Body Mass Index (BMI) is calculated as weight (in kilograms) divided by the square of height (in meters; kg/m^2^). According to the World Health Organization (WHO), BMI is categorized into four groups: underweight (less than 18.5 kg/m^2^), normal weight (18.5 to less than 25 kg/m^2^), overweight (25 to less than 30 kg/m^2^), and obese (30 kg/m^2^ or greater).^4^ Hypertension was defined as having an average systolic blood pressure greater than 140 mmHg, an average diastolic blood pressure greater than 90 mmHg, a history of being diagnosed with high blood pressure, or current use of prescription medication for high blood pressure, with at least one of these criteria being met. Diabetes status was determined based on a doctor’s diagnosis. Heart disease identification relied on four indicators: a history of congestive heart failure, coronary artery disease, angina pectoris, or a heart attack, with any one of these conditions affirming the presence of heart disease. The Urine Albumin to Creatinine Ratio (UACR) was categorized as <30 mg/g for normal individuals and ≥ 30 mg/g indicating abnormal proteinuria. Glycosylated hemoglobin (HbA1c) levels were also considered, serving as an indicator of long-term glycemic control.

### 2.5 Statistical analysis

To obtain more representative estimates, this study applied the MEC weights recommended by the NHANES database in all analyses. All data collection and statistical analyses were performed using R4.2.0^5^ and Empower Stats.^6^

The relationship between dietary folate intake and serum Klotho levels was examined using multivariate linear regression analysis, which facilitated the computation of beta values and 95% confidence intervals. This analysis was structured into three distinct models: Model 1, which did not adjust for any variables; Model 2, which adjusted for sex, age, and race; and Model 3, which incorporated adjustments for additional covariates. Serum Klotho levels were analyzed both as a continuous independent variable and categorically, divided into quartiles, to evaluate trends. To visually represent this relationship, a smoothed curve-fitting model was employed. The study also conducted stratified and interaction analyses to investigate how this association might differ across subgroups defined by each covariate. Specifically, analyses were stratified by age (comparing those under 60 years to those 60 years and older), sex, and the presence of hypertension across varying dietary folate intake.

Further, sensitivity analyses were carried out on distinct population subsets: those consuming only naturally occurring folate in foods, those whose intake included only dietary folic acid from fortified food [calculated as μg of DFEs provided = μg of natural food folate + (1.7 × μg of folic acid)] (32), and those consuming folate exclusively through supplements. The characteristics of these populations are detailed in Supplementary Table S1. These analyses aimed to assess the impact of potential outliers on the findings. To ensure the stability of the outcomes, the study excluded data points representing extreme values, this included individuals with Klotho levels above 2,500 pg./mL, those with dietary folate intake exceeding 500 μg/1000 kcal or below 100 μg/1000 kcal after energy adjustment, and individuals with energy-adjusted vitamin B12 levels greater than 6.0 μg/1000 kcal. Additionally, individuals categorized as “obese” by BMI, as well as those diagnosed with hypertension, diabetes, or heart disease, and those with a urine albumin/creatinine ratio (UACR) greater than 30 mg/g were also excluded.

## 3 Results

### 3.1 Baseline characteristics of all participants

This study enrolled a total of 10,278 participants, of whom 47.75% were male and 52.25% were female. The age range of participants spanned from 40 to 79 years, with a mean age of 57.64 years (standard deviation ±10.82 years). Participants were stratified based on the quartiles of serum Klotho levels, and the baseline characteristics are delineated in Table 1. Significant differences were observed across the quartiles of Dietary folate intake in terms of age, gender, race, marital status, Poor income ratio, body mass index (BMI), diabetes mellitus, urinary albumin-to-creatinine ratio (UACR), and glycosylated hemoglobin levels.

**Table 1:** Characteristic of the study population in NHANES 2007–2016.

		Dietary folate intake (μg/1000 kcal)				
Variables	Total	Q1 (41.83–147.61)	Q2 (147.62–186.00)	Q3 (186.03–239.11)	Q4 (239.2–1291.04)	*p*-value
N (%)	10,278	2,569 (25.00%)	2,570 (25.00%)	2,569 (25.00%)	2,570 (25.00%)	
Serum Klotho quartiles (pg/mL)	856.03 ± 311.67	846.83 ± 327.15	860.78 ± 320.93	856.29 ± 301.48	860.22 ± 296.04	0.018
Age (years)	57.64 ± 10.82	56.11 ± 10.54	57.47 ± 10.80	58.25 ± 10.97	58.73 ± 10.81	<0.001
UACR(mg/g)	47.67 ± 359.83	48.62 ± 374.18	41.86 ± 286.60	53.26 ± 398.82	46.95 ± 369.89	0.046
HbA1c (%)	5.94 ± 1.15	5.94 ± 1.19	5.94 ± 1.16	5.94 ± 1.15	5.94 ± 1.10	0.953
Gender (%)						<0.001
Male	4,908 (47.75%)	1,371 (53.37%)	1,221 (47.51%)	1,156 (45.00%)	1,160 (45.14%)	
Female	5,370 (52.25%)	1,198 (46.63%)	1,349 (52.49%)	1,413 (55.00%)	1,410 (54.86%)	
Ethnicity/Race (%)						<0.001
Non-Hispanic White	1,486 (14.46%)	314 (12.22%)	388 (15.10%)	404 (15.73%)	380 (14.79%)	
Non-Hispanic Black	1,096 (10.66%)	194 (7.55%)	264 (10.27%)	321 (12.50%)	317 (12.33%)	
Mexican American	4,832 (47.01%)	1,226 (47.72%)	1,228 (47.78%)	1,168 (45.47%)	1,210 (47.08%)	
Other Race - Including Multi-Racial	2001 (19.47%)	721 (28.07%)	535 (20.82%)	415 (16.15%)	330 (12.84%)	
Other Hispanic	863 (8.40%)	114 (4.44%)	155 (6.03%)	261 (10.16%)	333 (12.96%)	
Marital (%)						<0.001
Married	6,260 (60.91%)	1,468 (57.14%)	1,541 (59.96%)	1,596 (62.13%)	1,655 (64.40%)	
Currently in a relationship	478 (4.65%)	155 (6.03%)	128 (4.98%)	106 (4.13%)	89 (3.46%)	
Not currently in a partner	3,540 (34.44%)	946 (36.82%)	901 (35.06%)	867 (33.75%)	826 (32.14%)	
Poor income ratio (%)						<0.001
<1.3	2,896 (28.18%)	784 (30.52%)	735 (28.60%)	673 (26.20%)	704 (27.39%)	
1.3–3.5	3,740 (36.39%)	983 (38.26%)	954 (37.12%)	915 (35.62%)	888 (34.55%)	
≥3.5	3,642 (35.43%)	802 (31.22%)	881 (34.28%)	981 (38.19%)	978 (38.05%)	
BMI (%)						<0.001
Underweight (<18.5 kg/m2)	101 (0.98%)	32 (1.25%)	25 (0.97%)	28 (1.09%)	16 (0.62%)	
Normal weight (18.5–24.9 kg/m2)	2,323 (22.60%)	487 (18.96%)	542 (21.09%)	627 (24.41%)	667 (25.95%)	
Overweight (25–29.9 kg/m2)	3,496 (34.01%)	849 (33.05%)	875 (34.05%)	872 (33.94%)	900 (35.02%)	
Obese (≥30 kg/m2)	4,358 (42.40%)	1,201 (46.75%)	1,128 (43.89%)	1,042 (40.56%)	987 (38.40%)	
Hypertension (%)						0.433
No	5,524 (53.75%)	1,404 (54.65%)	1,363 (53.04%)	1,399 (54.46%)	1,358 (52.84%)	
Yes	4,754 (46.25%)	1,165 (45.35%)	1,207 (46.96%)	1,170 (45.54%)	1,212 (47.16%)	
Diabetes (%)						0.009
Yes	1769 (17.21%)	384 (14.95%)	454 (17.67%)	447 (17.40%)	484 (18.83%)	
No	8,206 (79.84%)	2,113 (82.25%)	2032 (79.07%)	2054 (79.95%)	2007 (78.09%)	
Borderline	303 (2.95%)	72 (2.80%)	84 (3.27%)	68 (2.65%)	79 (3.07%)	
CVD (%)						0.115
No	8,970 (87.27%)	2,235 (87.00%)	2,243 (87.28%)	2,274 (88.52%)	2,218 (86.30%)	
Yes	1,308 (12.73%)	334 (13.00%)	327 (12.72%)	295 (11.48%)	352 (13.70%)	

Mean +/− SD for: age, dietary folate intake, UACR, HbA1c. % for: gender, race, marital, poor income ratio, BMI, hypertension, diabetes, CVD.

### 3.2 Association between dietary folate intake and serum klotho levels

In the multivariate regression analysis detailed in Table 2, an association was observed between dietary folate intake and serum Klotho levels. Specifically, in both Model 2 and Model 3, each 1 μg/1000 kcal increase in energy-adjusted dietary folate intake was associated with a 0.11 pg./mL increase in serum Klotho levels. Subgroup analyses in Models 2 and 3 showed significant associations for Groups Q2, Q3, and Q4 compared to Group Q1, with *p*-values less than 0.05 for each group. The trend difference between the groups in both Model 2 and Model 3 was significant, with *p*-values for trend less than 0.0022 and 0.0035, respectively.

**Table 2:** Association between dietary folate intake and serum klotho levels among adults in NHANES 2007–2016.

Dietary folate intake (μg/1000 kcal)	Model 1		Model 2		Model 3	
	β (95%CI)	*p*-value	β (95%CI)	*p*-value	β (95%CI)	*p*-value
Continuous	0.09 (0.02, 0.15)	0.0079	0.11 (0.05, 0.18)	0.0005	0.11 (0.05, 0.18)	0.0009
Quartiles						
Q1(41.83–147.61)	Reference		Reference		Reference	
Q2(147.62–186.00)	27.34 (11.27, 43.41)	0.0009	30.58 (14.59, 46.57)	0.0002	29.79 (13.84, 45.75)	0.0003
Q3(186.03–239.11)	18.69 (2.54, 34.84)	0.0234	23.36 (7.18, 39.53)	0.0047	21.17 (5.01, 37.34)	0.0103
Q4(239.2–1291.04)	24.17 (8.05, 40.29)	0.0033	31.13 (14.96, 47.30)	0.0002	30.15 (13.94, 46.37)	0.0003
*p* for trend		0.0240		0.0022		0.0035

Model 1: no cofounder. Model 2: adjusted for age, gender, race. Model 3: adjusted for age, gender, race, marital, BMI, poor income ratio, hypertension, diabetes, CVD, HbA1c and UACR. CI, confidence interval.

**Figure 2:**
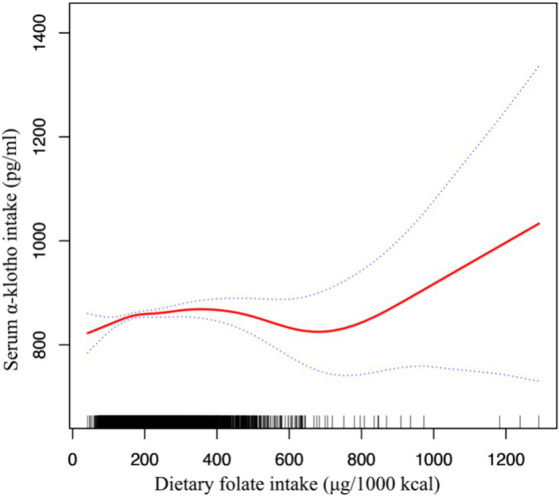
Association between dietary folate intake and serum Klotho levels adjusted for age, gender, race, marital, body mass index, poor income ratio, hypertension, diabetes, CVD, HbA1c and UACR.

We used smoothed curve fitting to show the complex, non-linear relationship between folate intake and Klotho levels in the blood (Figure 2). This technique helps highlight how these two variables interact beyond straightforward linear patterns.

### 3.3 Subgroup analysis and sensitivity analysis

In the subgroup analysis examining the relationship between dietary folate intake and Klotho levels (Table 3), the study population was stratified into quartiles based on their dietary folate intake. Following adjustments for potential confounding variables, the correlation appeared to be more pronounced among individuals aged 60 years or older, within the male demographic, and among participants with a history of hypertension.

**Table 3:** Stratified analyses of association between dietary folate intake and serum klotho levels in NHANES 2007–2016.

Variable	Dietary folate intake (μg/1000 kcal), β (95%CI)		*p*-value	*P* for interaction
Age subgroup				0.0742
<60 years	41.83–147.61	Reference		
	147.62–186.00	8.76 (−13.09, 30.60)	0.4321	
	186.03–239.11	11.92 (−10.58, 34.42)	0.2992	
	239.2–1291.04	16.46 (−6.04, 38.95)	0.1517	
	*P for trend*		0.1614	
≥60 years	41.83–147.61	Reference		
	147.62–186.00	64.30 (41.09, 87.51)	<0.0001	
	186.03–239.11	38.36 (15.52, 61.20)	0.0010	
	239.2–1291.04	51.37 (28.36, 74.39)	<0.0001	
	*P for trend*		0.0036	
Gender subgroup				0.1776
Male	41.83–147.61	Reference		
	147.62–186.00	26.54 (5.60, 47.48)	0.0130	
	186.03–239.11	3.29 (−18.37, 24.94)	0.7660	
	239.2–1291.04	37.55 (15.88, 59.21)	0.0007	
	*P for trend*		0.0047	
Female	41.83–147.61	Reference		
	147.62–186.00	33.65 (9.66, 57.64)	0.0060	
	186.03–239.11	37.52 (13.60, 61.45)	0.0021	
	239.2–1291.04	26.73 (2.74, 50.71)	0.0290	
	*P for trend*		0.1116	
Hypertension subgroup				0.3968
No	41.83–147.61	Reference		
	147.62–186.00	49.02 (27.29, 70.75)	<0.0001	
	186.03–239.11	27.55 (5.67, 49.44)	0.0136	
	239.2–1291.04	30.01 (7.83, 52.19)	0.0080	
	*P for trend*		0.1055	
Yes	41.83–147.61	Reference		
	147.62–186.00	−0.97 (−24.54, 22.60)	0.9357	
	186.03–239.11	9.40 (−14.69, 33.48)	0.4444	
	239.2–1291.04	26.95 (3.18, 50.72)	0.0263	
	*P for trend*		0.0114	

Adjusted for age, gender, race, marital, BMI, poor income ratio, hypertension, diabetes, CVD, HbA1c and UACR except the subgroup variable.

Furthermore, extensive sensitivity analyses were conducted in this study to assess the stability of the results (refer to Table 4): (1) only samples deriving from natural food folate intake were included; (2) Inclusion was limited to samples containing dietary folic acid from fortified foods; (3) the analysis included only samples with intake of folate supplements; (4) samples exhibiting Klotho levels >2,500 pg./mL were excluded; (5) Excluding samples with dietary folate intake exceeding 500 μg/1000 kcal; (6) exclusion of samples with <100 μg/1000 kcal of dietary folate intake; (7) samples with dietary vitamin B12 intake >6.0 μg/1000 kcal were also excluded; (8) given the potential influence of hyperlipidemia on Klotho levels, samples with a BMI categorization of “obese” were excluded (33); (9) considering the association of hypertension prevalence with lower Klotho levels, samples with a hypertension status of “yes” were excluded (34); (10) due to the nonlinear relationship between serum Klotho levels and the prevalence of diabetes mellitus, samples with a diabetes mellitus status of “yes” were excluded (35); (11) recognizing the significant correlations of serum Klotho with heart failure (36) and myocardial infarction (37), samples with a cardiac status of “yes” were excluded; (12) considering the association between Klotho levels and urinary albumin excretion rate (38) as well as chronic kidney disease albuminuria (15) samples with UACR results ≥30 were excluded. These results imply that folate intake from natural sources significantly impacts serum Klotho levels, whereas factors such as dietary fortification with folic acid and the prevalence of hypertension appear to have a negligible influence on this relationship.

**Table 4:** Inclusion and exclusion criteria.

	β (95% CI)	*p*-value
Only folate intake from natural foods was included (μg/1000 kcal)	0.24 (0.14, 0.34)	<0.0001
Only dietary folic acid from fortified food (DFE μg/1000 kcal)	0.02 (−0.06, 0.10)	0.7025
Only folic acid supplement intake was included (DFE μg/1000 kcal)	−0.01 (−0.03, 0.01)	0.4292
Samples with klotho level > 2,500 pg./mL were excluded	0.12 (0.06, 0.18)	0.0002
Samples with dietary folate intake >500 (μg/1000 kcal) were excluded	0.15 (0.07, 0.23)	0.0001
Samples with dietary folate intake <100 (μg/1000 kcal) were excluded	0.10 (0.03, 0.17)	0.0040
Samples with dietary vitb12 > 6.0 (μg/1000 kcal) were excluded	0.18 (0.10, 0.25)	<0.0001
Exclusion of obese people	0.13 (0.04, 0.21)	0.0032
Excluding people with hypertension	0.13 (0.04, 0.22)	0.0042
Exclusion of people with diabetes	0.13 (0.06, 0.20)	0.0004
Excluding people with heart disease	0.12 (0.05, 0.19)	0.0007
People with UACR≥30 mg/g were excluded	0.13 (0.06, 0.19)	0.0004

## 4 Discussion

In our study, we explored the relationship between dietary folate intake and serum Klotho levels, finding a nuanced interplay. This connection was stronger in individuals over 60, males, and those with hypertension, highlighting folate’s role in regulating the anti-aging factor Klotho. After adjusting for energy intake, each additional unit of dietary folate intake was associated with a 0.11 pg./mL increase in Klotho levels. This correlation may play a positive role in promoting healthy aging and preventing age-related diseases. Additionally, it lays the foundation for pioneering new health strategies focused on dietary interventions.

Folate intake has been found to exert significant effects on multiple signaling pathways, notably the mTOR pathway, the insulin/IGF-1 signaling pathway, and the Wnt/β-catenin signaling pathway. For instance, the research conducted by Fredrick J. Rosario and colleagues illustrates that maternal folate deficiency can impair fetal growth by inhibiting placental mTOR signaling, thereby establishing a direct connection between folate levels and mTOR signaling (39). Furthermore, Elena Silva’s studies have shown that folate deficiency influences cellular functions through innovative sensing mechanisms, which in turn activate the mTOR signaling pathway, affecting processes such as nutrient transport and protein synthesis (40). Additionally, Andrea Annibal and co-authors have discovered through high-resolution mass spectrometry analysis of the metabolome of the nematode Cryptomeria hidrobatidis, that one-carbon metabolism and the folate cycle are integral in jointly regulating lifespan, with insulin/IGF signaling playing a comparable regulatory role across different species’ longevity models. This suggests that certain metabolic nodes are pivotal in promoting healthy aging (41). Meanwhile, research by Wen-Chi L. Chang and others on the impact of folic acid supplementation in the development of colitis-associated colorectal cancer indicated that high doses of folic acid can encourage cancer formation by modifying the epigenetic field effects of the Wnt/β-catenin and MAPK signaling pathways (42). Despite the compelling evidence linking folate intake with mechanisms of anti-aging, a gap remains in the direct, controlled trials demonstrating a connection with Klotho, highlighting a promising avenue for future research.

The primary dietary sources of folate include green leafy vegetables (for instance, spinach and broccoli), citrus fruits, legumes, nuts, and whole grain products (43–47). To alleviate health issues caused by folate deficiency, since 1998, the United States and Canada have mandated the addition of dietary folic acid from fortified food to flour and grain products. Common foods containing fortified folic acid include flour, bread, breakfast cereals, pasta, and cornmeal (48–50). Our sensitivity analysis unveiled a noteworthy observation: a significant correlation was identified exclusively between folate intake from natural sources and serum Klotho levels, whereas folic acid obtained through fortified foods and supplements did not exhibit a similar association, paralleling the discoveries of Mengyi Liu et al. (51). This prompts us to suggest that there exists an “optimal” range for folate intake, with the intake of folate from natural foods generally falling precisely within this range (52). The excessive intake of folic acid may precipitate adverse effects, notably when surpassing the recommended daily allowance (53). Such overconsumption bears the risk of concealing the diagnosis of vitamin B12 deficiency and merely ameliorating anemia symptoms without addressing the potential for neurological harm (54–56). Furthermore, certain research suggests that a long-term, high-dose regimen of folic acid could correlate with an elevated risk of specific cancers (57). Consequently, it is imperative to regulate folic acid intake meticulously to prevent excessive ingestion (58).

Subgroup analyses have elucidated a broad decline in metabolic function and nutrient absorption capabilities as individuals age (59–61). Notably, research focusing on adults aged 60 and above has highlighted a correlation between folate intake and serum Klotho levels, particularly pronounced among those seniors with elevated folate intake (*p* < 0.0001, trend *p*-value = 0.0036). These findings underscore the significance of maintaining a moderate intake of folate within aging populations to support cardiovascular health, bone integrity, and other health facets (62, 63). Conversely, in individuals younger than 60 years, the link between folate intake and Klotho levels did not prove to be significant. This suggests that the demand for folate, along with its contribution to physical well-being, assumes greater importance as one ages, highlighting the potential benefits of targeted nutritional supplementation to meet the specific needs posed by aging (2, 26).

Moreover, we observed pronounced differences between genders regarding the association between dietary folate intake and serum Klotho levels among men and women (64). In men, a notable correlation was observed between folate intake and serum Klotho levels, particularly within the moderate to high folate intake category (*p*-values = 0.0007 and a trend p-value of 0.0047). Similarly, in females, dietary folate intake was significantly associated with serum Klotho levels (p-values ranging from 0.0021 to 0.0290, trend *p*-value = 0.1116). Although some literature suggests the potential influence of testosterone levels on Klotho protein expression in men (65–67), our current dataset does not directly investigate this hypothesis at either a physiological or molecular level. Thus, these findings underscore the critical need for subsequent research to more comprehensively explore how gender differences affect the interplay between folate intake and Klotho levels.

The potential beneficial impact of folate on the expression and functionality of Klotho proteins assumes critical importance, particularly in light of Klotho’s pivotal role in preserving vascular health and regulating the equilibrium of blood pressure (20, 62). This mode of action not only underscores the profound importance of folate in combating cardiovascular diseases but also illuminates its prospective utility in the realm of anti-aging (41).

This study is subject to certain limitations. Firstly, the NHANES cohort might not accurately reflect the global population, particularly in countries and regions where food is not fortified with folic acid. Thus, incorporating additional samples from a variety of centers could enhance the study’s validity. Secondly, like all dietary assessment methods, the 24-h dietary recall used in this study has inherent limitations. These include recall bias, as it relies on participants’ ability to accurately remember and report their food intake, and response bias, where participants might alter their reported intake to align with perceived social norms or to avoid negative judgment. Additionally, the 24-h recall provides a snapshot of a single day’s intake, which may not accurately represent usual dietary patterns. Moreover, while data from cross-sectional studies can establish associations, they are insufficient for determining causality, necessitating further evidence to elucidate the cause-and-effect relationship.

## 5 Conclusion

In summary, among a nationally representative population of American adults, our study identified a significant association between dietary folate intake and serum Klotho levels, especially prominent in men, individuals aged 60 and above, and those with hypertension. These findings suggest a potential role for dietary folate in supporting healthy aging and underscore the need for further research to understand its mechanisms and broader health implications.

## Data availability statement

Publicly available datasets were analyzed in this study. This data can be found at: https://wwwn.cdc.gov/nchs/nhanes/Default.aspx.

## Ethics statement

The studies involving humans were approved by NCHS Ethics Review Board (ERB) Approval. The studies were conducted in accordance with the local legislation and institutional requirements. The participants provided their written informed consent to participate in this study.

## Author contributions

YL: Data curation, Writing – original draft, Writing – review & editing. CZ: Conceptualization, Writing – review & editing. RS: Data curation, Writing – review & editing. AW: Conceptualization, Writing – review & editing. TZ: Writing – review & editing. ZC: Conceptualization, Writing – review & editing.

## Footnotes

^1^https://www.cdc.gov/nchs/nhanes/about_nhanes.htm

^2^http://www.ars.usda.gov/ba/bhnrc/fsrg

^3^https://aspe.hhs.gov/prior-hhs-poverty-guidelines-and-federal-register-references

^4^https://www.who.int/data/gho/data/themes/topics/topic-details/GHO/body-mass-index

^5^http://www.r-project.org

^6^http://www.empowerstats.com

## References

[1] Bjørklund G Shanaida M Lysiuk R Butnariu M Peana M Sarac I. Natural compounds and products from an anti-aging perspective. Molecules. (2022) 27:7084. doi: 10.3390/molecules27207084, PMID: 36296673 PMC9610014

[2] Yousefzadeh M Henpita C Vyas R Soto-Palma C Robbins P Niedernhofer L. DNA damage-how and why we age? eLife. (2021) 10:10. doi: 10.7554/eLife.62852 PMC7846274 33512317

[3] Benayoun BA Pollina EA Brunet A. Epigenetic regulation of ageing: linking environmental inputs to genomic stability. Nat Rev Mol Cell Biol. (2015) 16:593–610. doi: 10.1038/nrm4048, PMID: 26373265 PMC4736728

[4] Castaldi A Dodia RM Orogo AM Zambrano CM Najor RH Gustafsson ÅB. Decline in cellular function of aged mouse C-kit(+) cardiac progenitor cells. J Physiol. (2017) 595:6249–62. doi: 10.1113/jp274775, PMID: 28737214 PMC5621489

[5] Schumacher B Pothof J Vijg J Hoeijmakers JHJ. The central role of DNA damage in the ageing process. Nature. (2021) 592:695–703. doi: 10.1038/s41586-021-03307-7, PMID: 33911272 PMC9844150

[6] Johnson AA Cuellar TL. Glycine and aging: evidence and mechanisms. Ageing Res Rev. (2023) 87:101922. doi: 10.1016/j.arr.2023.101922, PMID: 37004845

[7] Kuro-o M Matsumura Y Aizawa H Kawaguchi H Suga T Utsugi T. Mutation of the mouse klotho gene leads to a syndrome resembling ageing. Nature. (1997) 390:45–51. doi: 10.1038/36285, PMID: 9363890

[8] Abraham CR Li A. Aging-suppressor klotho: prospects in diagnostics and therapeutics. Ageing Res Rev. (2022) 82:101766. doi: 10.1016/j.arr.2022.101766, PMID: 36283617

[9] Buchanan S Combet E Stenvinkel P Shiels PG. Klotho, aging, and the failing kidney. Front Endocrinol (Lausanne). (2020) 11:560. doi: 10.3389/fendo.2020.00560, PMID: 32982966 PMC7481361

[10] Semba RD Moghekar AR Hu J Sun K Turner R Ferrucci L. Klotho in the cerebrospinal fluid of adults with and without Alzheimer's disease. Neurosci Lett. (2014) 558:37–40. doi: 10.1016/j.neulet.2013.10.058, PMID: 24211693 PMC4037850

[11] Miao J Liu J Niu J Zhang Y Shen W Luo C. Wnt/Β-catenin/Ras signaling mediates age-related renal fibrosis and is associated with mitochondrial dysfunction. Aging Cell. (2019) 18:e13004. doi: 10.1111/acel.13004, PMID: 31318148 PMC6718575

[12] Sopjani M Rinnerthaler M Kruja J Dermaku-Sopjani M. Intracellular signaling of the aging suppressor protein klotho. Curr Mol Med. (2015) 15:27–37. doi: 10.2174/1566524015666150114111258, PMID: 25601466

[13] Chen K Wang S Sun QW Zhang B Ullah M Sun Z. Klotho deficiency causes heart aging via impairing the Nrf2-gr pathway. Circ Res. (2021) 128:492–507. doi: 10.1161/circresaha.120.317348, PMID: 33334122 PMC8782577

[14] Torres PU Prié D Molina-Blétry V Beck L Silve C Friedlander G. Klotho: an antiaging protein involved in mineral and vitamin D metabolism. Kidney Int. (2007) 71:730–7. doi: 10.1038/sj.ki.5002163 17332731

[15] Zhang J Zhang A. Relationships between serum klotho concentrations and cognitive performance among older chronic kidney disease patients with albuminuria in Nhanes 2011-2014. Front Endocrinol (Lausanne). (2023) 14:1215977. doi: 10.3389/fendo.2023.1215977, PMID: 37560310 PMC10407554

[16] Zhou H Pu S Zhou H Guo Y. Klotho as potential autophagy regulator and therapeutic target. Front Pharmacol. (2021) 12:755366. doi: 10.3389/fphar.2021.755366, PMID: 34737707 PMC8560683

[17] Yi YY Chen H Zhang SB Xu HW Fang XY Wang SJ. Exogenous klotho ameliorates extracellular matrix degradation and angiogenesis in intervertebral disc degeneration via inhibition of the Rac1/Pak1/Mmp-2 signaling Axis. Mech Ageing Dev. (2022) 207:111715. doi: 10.1016/j.mad.2022.111715, PMID: 35952859

[18] Castner SA Gupta S Wang D Moreno AJ Park C Chen C. Longevity factor klotho enhances cognition in aged nonhuman Primates. Nat Aging. (2023) 3:931–7. doi: 10.1038/s43587-023-00441-x, PMID: 37400721 PMC10432271

[19] Kale A Sankrityayan H Anders HJ Gaikwad AB. Epigenetic and non-epigenetic regulation of klotho in kidney disease. Life Sci. (2021) 264:118644. doi: 10.1016/j.lfs.2020.118644, PMID: 33141039

[20] Zheng Y Cantley LC. Toward a better understanding of folate metabolism in health and disease. J Exp Med. (2019) 216:253–66. doi: 10.1084/jem.20181965, PMID: 30587505 PMC6363433

[21] Clare CE Brassington AH Kwong WY Sinclair KD. One-carbon metabolism: linking nutritional biochemistry to epigenetic programming of long-term development. Annu Rev Anim Biosci. (2019) 7:263–87. doi: 10.1146/annurev-animal-020518-115206, PMID: 30412672

[22] Koury MJ Ponka P. New insights into erythropoiesis: the roles of folate, vitamin B12, and Iron. Annu Rev Nutr. (2004) 24:105–31. doi: 10.1146/annurev.nutr.24.012003.132306, PMID: 15189115

[23] Stanhewicz AE Kenney WL. Role of folic acid in nitric oxide bioavailability and vascular endothelial function. Nutr Rev. (2017) 75:61–70. doi: 10.1093/nutrit/nuw053, PMID: 27974600 PMC5155615

[24] Copp AJ Greene ND. Neural tube defects: prevention by folic acid and other vitamins. Indian J Pediatr. (2000) 67:915–21. doi: 10.1007/bf02723958 11262991

[25] Tamura T Picciano MF. Folate and human reproduction. Am J Clin Nutr. (2006) 83:993–1016. doi: 10.1093/ajcn/83.5.993 16685040

[26] Liu Y Geng T Wan Z Lu Q Zhang X Qiu Z. Associations of serum folate and vitamin B12 levels with cardiovascular disease mortality among patients with type 2 diabetes. JAMA Netw Open. (2022) 5:e2146124. doi: 10.1001/jamanetworkopen.2021.46124, PMID: 35099545 PMC8804919

[27] Ashraf MJ Cook JR Rothberg MB. Clinical utility of folic acid testing for patients with Anemia or dementia. J Gen Intern Med. (2008) 23:824–6. doi: 10.1007/s11606-008-0615-z, PMID: 18414954 PMC2517884

[28] Paniz C Bertinato JF Lucena MR De Carli E Amorim P Gomes GW. A daily dose of 5 mg folic acid for 90 days is associated with increased serum Unmetabolized folic acid and reduced natural killer cell cytotoxicity in healthy Brazilian adults. J Nutr. (2017) 147:1677–85. doi: 10.3945/jn.117.247445, PMID: 28724658 PMC5712455

[29] Wu SE Chen YJ Chen WL. Adherence to Mediterranean diet and soluble klotho level: the value of food synergy in aging. Nutrients. (2022) 14:3910. doi: 10.3390/nu14193910, PMID: 36235560 PMC9573612

[30] Liu S Wu M Wang Y Xiang L Luo G Lin Q. The association between dietary Fiber intake and serum klotho levels in Americans: a cross-sectional study from the National Health and nutrition examination survey. Nutrients. (2023) 15:3147. doi: 10.3390/nu15143147, PMID: 37513564 PMC10385840

[31] Willett WC Howe GR Kushi LH. Adjustment for Total energy intake in epidemiologic studies. Am J Clin Nutr. (1997) 65:1220S–8S. doi: 10.1093/ajcn/65.4.1220S 9094926

[32] Institute of Medicine Standing Committee on the Scientific Evaluation of Dietary Reference I, its Panel on Folate OBV, Choline. Dietary Reference Intakes for Thiamin, Riboflavin, Niacin, Vitamin B(6), Folate, Vitamin B(12), Pantothenic Acid, Biotin, and Choline. Washington (DC): National Academies Press (1998). 23193625

[33] Yan S Luo W Lei L Zhang Q Xiu J. Association between serum klotho concentration and hyperlipidemia in adults: a cross-sectional study from Nhanes 2007-2016. Front Endocrinol (Lausanne). (2023) 14:1280873. doi: 10.3389/fendo.2023.1280873, PMID: 38027194 PMC10668332

[34] Yan Y Chen J. Association between serum klotho concentration and all-cause and cardiovascular mortality among American individuals with hypertension. Front Cardiovasc Med. (2022) 9:1013747. doi: 10.3389/fcvm.2022.1013747, PMID: 36457804 PMC9705974

[35] Wang K Mao Y Lu M Liu X Sun Y Li Z. Association between serum klotho levels and the prevalence of diabetes among adults in the United States. Front Endocrinol (Lausanne). (2022) 13:1005553. doi: 10.3389/fendo.2022.1005553, PMID: 36440221 PMC9681912

[36] Cai J Zhang L Chen C Ge J Li M Zhang Y. Association between serum klotho concentration and heart failure in adults, a cross-sectional study from Nhanes 2007-2016. Int J Cardiol. (2023) 370:236–43. doi: 10.1016/j.ijcard.2022.11.010, PMID: 36351541

[37] Xu JP Zeng RX He MH Lin SS Guo LH Zhang MZ. Associations between serum soluble Α-klotho and the prevalence of specific cardiovascular disease. Front Cardiovasc Med. (2022) 9:899307. doi: 10.3389/fcvm.2022.899307, PMID: 35795366 PMC9251131

[38] Chang K Li Y Qin Z Zhang Z Wang L Yang Q. Association between serum soluble Α-klotho and urinary albumin excretion in middle-aged and older us adults: Nhanes 2007-2016. J Clin Med. (2023) 12:637. doi: 10.3390/jcm12020637, PMID: 36675565 PMC9863467

[39] Rosario FJ Nathanielsz PW Powell TL Jansson T. Maternal folate deficiency causes inhibition of Mtor signaling, Down-regulation of placental amino acid transporters and fetal growth restriction in mice. Sci Rep. (2017) 7:3982. doi: 10.1038/s41598-017-03888-2, PMID: 28638048 PMC5479823

[40] Silva E Rosario FJ Powell TL Jansson T. Mechanistic target of rapamycin is a novel molecular mechanism linking folate availability and cell function. J Nutr. (2017) 147:1237–42. doi: 10.3945/jn.117.248823, PMID: 28592519 PMC5483964

[41] Annibal A Tharyan RG Schonewolff MF Tam H Latza C Auler MMK. Regulation of the one carbon folate cycle as a shared metabolic signature of longevity. Nat Commun. (2021) 12:3486. doi: 10.1038/s41467-021-23856-9, PMID: 34108489 PMC8190293

[42] Chang WL Ghosh J Cooper HS Vanderveer L Schultz B Zhou Y. Folic acid supplementation promotes Hypomethylation in both the inflamed colonic mucosa and colitis-associated dysplasia. Cancers (Basel). (2023) 15:2949. doi: 10.3390/cancers15112949, PMID: 37296911 PMC10252136

[43] Morris MC Wang Y Barnes LL Bennett DA Dawson-Hughes B Booth SL. Nutrients and bioactives in green leafy vegetables and cognitive decline: prospective study. Neurology. (2018) 90:e214–22. doi: 10.1212/wnl.0000000000004815, PMID: 29263222 PMC5772164

[44] Saini RK Nile SH Park SW. Carotenoids from fruits and vegetables: chemistry, analysis, occurrence, bioavailability and biological activities. Food Res Int. (2015) 76:735–50. doi: 10.1016/j.foodres.2015.07.047, PMID: 28455059

[45] Miles EA Calder PC. Effects of Citrus fruit juices and their bioactive components on inflammation and immunity: a narrative review. Front Immunol. (2021) 12:712608. doi: 10.3389/fimmu.2021.712608, PMID: 34249019 PMC8264544

[46] Thomas PM Flanagan VP Pawlosky RJ. Determination of 5-Methyltetrahydrofolic acid and folic acid in Citrus juices using stable isotope dilution-mass spectrometry. J Agric Food Chem. (2003) 51:1293–6. doi: 10.1021/jf020902e, PMID: 12590471

[47] Liang Q Wang K Shariful I Ye X Zhang C. Folate content and retention in wheat grains and wheat-based foods: effects of storage, processing, and cooking methods. Food Chem. (2020) 333:127459. doi: 10.1016/j.foodchem.2020.127459, PMID: 32683256

[48] Crider KS Bailey LB Berry RJ. Folic acid food fortification-its history, effect, concerns, and future directions. Nutrients. (2011) 3:370–84. doi: 10.3390/nu3030370, PMID: 22254102 PMC3257747

[49] Centeno Tablante E Pachón H Guetterman HM Finkelstein JL. Fortification of wheat and maize flour with folic acid for population health outcomes. Cochrane Database Syst Rev. (2019) 7:CD012150. doi: 10.1002/14651858.CD012150.pub2, PMID: 31257574 PMC6599881

[50] Wang A Rose CE Qi YP Williams JL Pfeiffer CM Crider KS. Impact of voluntary folic acid fortification of corn Masa flour on Rbc folate concentrations in the U.S. (Nhanes 2011-2018). Nutrients. (2021) 13:1325. doi: 10.3390/nu13041325, PMID: 33923768 PMC8073626

[51] Liu M Ye Z Yang S Zhang Y Zhang Y He P. Relationship of dietary intake of food folate and synthetic folic acid intake from fortified foods with all-cause mortality in individuals with chronic kidney disease. Food Funct. (2024) 15:559–68. doi: 10.1039/d3fo03927g, PMID: 38164661

[52] Gomes S Lopes C Pinto E. Folate and folic acid in the Periconceptional period: recommendations from official health organizations in thirty-six countries worldwide and who. Public Health Nutr. (2016) 19:176–89. doi: 10.1017/s1368980015000555, PMID: 25877429 PMC10270901

[53] Patel KR Sobczyńska-Malefora A. The adverse effects of an excessive folic acid intake. Eur J Clin Nutr. (2017) 71:159–63. doi: 10.1038/ejcn.2016.194 27731331

[54] Henry CJ Nemkov T Casás-Selves M Bilousova G Zaberezhnyy V Higa KC. Folate dietary insufficiency and folic acid supplementation similarly impair metabolism and compromise hematopoiesis. Haematologica. (2017) 102:1985–94. doi: 10.3324/haematol.2017.171074, PMID: 28883079 PMC5709097

[55] Zhao G Deng J Shen Y Zhang P Dong H Xie Z. Hyperhomocysteinemia is key for increased susceptibility to Pnd in aged mice. Ann Clin Transl Neurol. (2019) 6:1435–44. doi: 10.1002/acn3.50838, PMID: 31353838 PMC6689684

[56] Gil Martínez V Avedillo Salas A Santander BS. Vitamin supplementation and dementia: a systematic review. Nutrients. (2022) 14:1033. doi: 10.3390/nu14051033, PMID: 35268010 PMC8912288

[57] Vegrim HM Dreier JW Alvestad S Gilhus NE Gissler M Igland J. Cancer risk in children of mothers with epilepsy and high-dose folic acid use during pregnancy. JAMA Neurol. (2022) 79:1130–8. doi: 10.1001/jamaneurol.2022.2977, PMID: 36156660 PMC9513705

[58] Fardous AM Heydari AR. Uncovering the hidden dangers and molecular mechanisms of excess folate: a narrative review. Nutrients. (2023) 15:4699. doi: 10.3390/nu15214699, PMID: 37960352 PMC10648405

[59] Mattson MP Arumugam TV. Hallmarks of brain aging: adaptive and pathological modification by metabolic states. Cell Metab. (2018) 27:1176–99. doi: 10.1016/j.cmet.2018.05.011, PMID: 29874566 PMC6039826

[60] Xie S Xu SC Deng W Tang Q. Metabolic landscape in cardiac aging: insights into molecular biology and therapeutic implications. Signal Transduct Target Ther. (2023) 8:114. doi: 10.1038/s41392-023-01378-8, PMID: 36918543 PMC10015017

[61] López-Otín C Blasco MA Partridge L Serrano M Kroemer G. The hallmarks of aging. Cell. (2013) 153:1194–217. doi: 10.1016/j.cell.2013.05.039, PMID: 23746838 PMC3836174

[62] Zhou YH Tang JY Wu MJ Lu J Wei X Qin YY. Effect of folic acid supplementation on cardiovascular outcomes: a systematic review and Meta-analysis. PLoS One. (2011) 6:e25142. doi: 10.1371/journal.pone.0025142 21980387 PMC3182189

[63] Fratoni V Brandi ML. B vitamins, homocysteine and bone health. Nutrients. (2015) 7:2176–92. doi: 10.3390/nu7042176, PMID: 25830943 PMC4425139

[64] Espuch-Oliver A Vázquez-Lorente H Jurado-Fasoli L de Haro-Muñoz T Díaz-Alberola I López-Velez MDS. References values of soluble Α-klotho serum levels using an enzyme-linked immunosorbent assay in healthy adults aged 18-85 years. J Clin Med. (2022) 11:2415. doi: 10.3390/jcm11092415, PMID: 35566540 PMC9101232

[65] Hsu SC Huang SM Lin SH Ka SM Chen A Shih MF. Testosterone increases renal anti-aging klotho gene expression via the androgen receptor-mediated pathway. Biochem J. (2014) 464:221–9. doi: 10.1042/bj20140739, PMID: 25163025

[66] Dote-Montero M Amaro-Gahete FJ De-la OA Jurado-Fasoli L Gutierrez A Castillo MJ. Study of the Association of Dheas, testosterone and cortisol with S-klotho plasma levels in healthy sedentary middle-aged adults. Exp Gerontol. (2019) 121:55–61. doi: 10.1016/j.exger.2019.03.010, PMID: 30928678

[67] Zhang Z Qiu S Huang X Jin K Zhou X Lin T. Association between testosterone and serum soluble Α-klotho in U.S. males: a cross-sectional study. BMC Geriatr. (2022) 22:570. doi: 10.1186/s12877-022-03265-3, PMID: 35820842 PMC9275159

